# Supplementary material for: Clothing the Emperor: Dynamic Root–Shoot Allocation Trajectories in Relation to Whole-Plant Growth Rate and in Response to Temperature
Source: Plants (Basel). 2019 Jul 10;8(7):212. doi: 10.3390/plants8070212 (PMC6681223; doi:10.3390/plants8070212)
Supplement: Supplementary file 1 [file plants-08-00212-s001.pdf]

Table S1. Criteria used for the selection of species.

Species	Guild	Life history	Life-form	Established strategy	Canopy structure	Canopy height	Lateral spread	Leaf phenology	Root system	Root depth	Seedling RGR	Main habitat type	Soil pH
Anthoxanthum odoratum	G	P	H	SR/CSR	L	2	2	Ea	A	S	S	P	5
Arrhenatherum elatius	G	P	H	C/CSR	L	5	4	Ep	A	D	I	Sk	7
*Catapodium rigidum**	G	Aws	T	SR	S	1	1	Sh	A	S	I	Sp	7
Deschampsia flexuosa	G	P	H	S/SC	S	2	4	Ea	A	S	S	P	3
Festuca ovina	G	P	H	S	S	2	3	Ea	A	S	S	P	W
Holcus lanatus	G	P	H	CSR	L	3	3	Ep	A	S/D	F	P/Sp	5
Poa annua	G	As	T/H	R	S	2	1	Ep	A	S	F	A	7
Cardamine hirsuta	F	Aws	T	SR	S	2	1	Sh	T	S	nd	Sp	7
Centaurea nigra	F	P	H	S/CSR	S	3	2	Sa	T	D	I	W	7
Rumex acetosa	F	P	H	CSR	S	2	2	Ea	T	D	I	P	5
Scabiosa columbaria	F	M/P	H	S/SR	S	1	2	Ea	T	D	I	P/Sk	7

*Listed [29] as *Desmazeria rigida* (L.) Tutin; nd = no data

Species were selected [25] to include examples which differed in a range of ecological, morphological and physiological features. These were based largely on information from standardised lab screening programmes, field surveys and literature reviews compiled later [29]. The final choice also depended on the availability of seed, the sources of which were from wild collections except for *Poa annua*, which was a commercial variety known to be annual.

Table S1 continued.

Key:	
Guild	G = grass; F = forb
Life history	As = summer annual; Aws = summer or winter annual; M = monocarpic perennial; P = polycarpic perennial
Life-form	H = hemicryptophyte (buds at soil level); T = therophyte (plant passing unfavourable season as seeds)
Established strategy	C = competitor; R = ruderal; S = stress-tolerator; SC = stress-tolerant competitor; SR = stress-tolerant ruderal; CSR = competitive stress-tolerant ruderal [51]
Canopy structure	L = leafy (no basal rosette); S = semi-rosette
Canopy height	1 = foliage <100 mm tall; 2 = 101-299 mm; 3 = 300-599 mm; 4 = 600-699 mm; 5 = 1-3 m
Lateral spread	1 = therophytes (limited spread); 2 = perennial with compact rhizomes/tussocks <100 mm diameter; 3 = perennials with rhizomes/tussocks 100-230 mm; 4 = perennials 251-1000 mm
Leaf phenology	Ea = canopy always evergreen; Ep = partially evergreen (species evergreen in only some habitats, in only mild winters, or leaves only partially senescent over winter); Sa = seasonal, canopy from spring to autumn; Sh = seasonal, mainly autumn to early summer
Root system	A = adventitious; T = taproot
Root depth	D = deep; S = shallow
Seedling RGR	As determined in laboratory screening tests: S = slow (RGR 0.07-0.13 d^{-1}); I = intermediate (0.14-0.20); F = fast (0.21-0.27)
Main habitat type	Within the Sheffield region: A = arable; P = pasture; Sk = skeletal; Sp = spoil; W = wasteland
Soil pH	Within the Sheffield region: Number = modal soil pH class of sampled habitats; W = wide-ranging distribution

Table S2. Mean shoot and root dry weights.

Species	Time (d)	Control plants		Cooled plants	
		Shoot biomass (g)	Root biomass (g)	Shoot biomass (g)	Root biomass (g)
Anthoxanthum odoratum	7	0.002	0.001	0.002	0.001
	14	0.008	0.003	0.008	0.003
	21	0.029	0.012	0.029	0.012
	28	0.111	0.037	0.111	0.037
	35	0.346	0.102	0.239	0.087
	42	0.696	0.189	0.345	0.156
	49	1.334	0.352	0.520	0.279
	56	2.937	0.767	0.740	0.489
Arrhenatherum elatius	7	0.002	0.001	0.002	0.001
	14	0.010	0.003	0.010	0.003
	21	0.045	0.012	0.045	0.012
	28	0.179	0.047	0.179	0.047
	35	0.516	0.132	0.299	0.105
	42	1.104	0.277	0.436	0.171
	49	2.024	0.509	0.588	0.258
	56	4.648	1.171	0.826	0.416
Catapodium rigidum	7	0.001	0.0002	0.001	0.0002
	14	0.003	0.001	0.003	0.001
	21	0.009	0.003	0.009	0.003
	28	0.031	0.010	0.031	0.010
	35	0.071	0.023	0.062	0.019
	42	0.142	0.042	0.104	0.035
	49	0.342	0.099	0.199	0.071
	56	0.742	0.210	0.407	0.163
Deschampsia flexuosa	7	0.002	0.0009	0.002	0.0009
	14	0.005	0.0020	0.005	0.0020
	21	0.011	0.0041	0.011	0.0041
	28	0.026	0.0086	0.026	0.0086
	35	0.061	0.0165	0.051	0.0153
	42	0.094	0.0236	0.076	0.0262
	49	0.152	0.0385	0.102	0.0403
	56	0.235	0.0595	0.148	0.0664
Festuca ovina	7	0.001	0.0006	0.001	0.0006
	14	0.003	0.0014	0.003	0.0014
	21	0.009	0.0032	0.009	0.0032
	28	0.025	0.0076	0.025	0.0076
	35	0.063	0.0172	0.052	0.0138
	42	0.112	0.0273	0.087	0.0275
	49	0.184	0.0419	0.138	0.0470
	56	0.375	0.0826	0.225	0.0892

Table S2 continued

Species	Time (d)	Control plants		Cooled plants	
		Shoot biomass (g)	Root biomass (g)	Shoot biomass (g)	Root biomass (g)
Holcus lanatus	7	0.002	0.0006	0.002	0.0006
	14	0.009	0.0028	0.009	0.0028
	21	0.042	0.0125	0.042	0.0125
	28	0.184	0.0529	0.184	0.0529
	35	0.409	0.1025	0.378	0.1737
	42	0.866	0.2083	0.559	0.2768
	49	1.826	0.3770	0.831	0.4379
	56	3.831	0.6911	1.319	0.7585
Poa annua	7	0.001	0.0004	0.001	0.0004
	14	0.006	0.0019	0.006	0.0019
	21	0.030	0.0086	0.030	0.0086
	28	0.171	0.0458	0.171	0.0458
	35	0.445	0.1063	0.281	0.1044
	42	0.879	0.1796	0.504	0.1984
	49	2.017	0.3539	0.804	0.3250
	56	5.251	0.7833	1.345	0.5621
Cardamine hirsuta	7	0.002	0.0005	0.002	0.0005
	14	0.009	0.0015	0.009	0.0015
	21	0.042	0.0050	0.042	0.0050
	28	0.203	0.0160	0.203	0.0160
	35	0.471	0.0256	0.248	0.0209
	42	0.740	0.0486	0.366	0.0391
	49	1.124	0.0649	0.490	0.0607
	56	2.075	0.1263	0.654	0.0957
Centaurea nigra	7	0.004	0.0018	0.004	0.0018
	14	0.015	0.0059	0.015	0.0059
	21	0.049	0.0197	0.049	0.0197
	28	0.160	0.0629	0.160	0.0629
	35	0.461	0.1901	0.295	0.1652
	42	0.708	0.3193	0.424	0.2818
	49	1.205	0.5972	0.641	0.5024
	56	1.996	1.0832	0.870	0.7934
Rumex acetosa	7	0.004	0.0010	0.004	0.0010
	14	0.018	0.0042	0.018	0.0042
	21	0.057	0.0150	0.057	0.0150
	28	0.205	0.0600	0.205	0.0600
	35	0.526	0.1540	0.372	0.1255
	42	0.982	0.2935	0.513	0.1716
	49	1.658	0.4972	0.727	0.2379
	56	3.308	1.0667	1.034	0.3276

Table S2 continued

Species	Time (d)	Control plants		Cooled plants	
		**Shoot biomass (g)**	**Root biomass (g)**	**Shoot biomass (g)**	**Root biomass (g)**
Scabiosa columbaria	7	0.003	0.0010	0.003	0.0010
	14	0.012	0.0030	0.012	0.0030
	21	0.043	0.0092	0.043	0.0092
	28	0.141	0.0278	0.141	0.0278
	35	0.310	0.0583	0.193	0.0466
	42	0.655	0.1252	0.300	0.0878
	49	1.248	0.2384	0.398	0.1376
	56	2.792	0.5336	0.563	0.2387

Table S3. Fitted logistic models, correlations between RMF and RGR, and allometry.

(a) Control plants

Species	Logistic models (eqn. (1)) fitted to data (Table S2)						Time interval (d)	Correlation between RMF and RGR	Allometry	
	Shoot			Root						
	r (d^{-1})	Y_{max} (g)	R^2	r (d^{-1})	Y_{max} (g)	R^2			α	β
Anthoxanthum odoratum	0.148	7.68	0.996	0.132	2.83	0.996	7-56	0.86	0.90	0.27
							7-120	-0.06	0.98	0.33
Arrhenatherum elatius	0.159	10.9	0.994	0.156	2.88	0.994	7-56	0.96	0.99	0.25
							7-120	-0.26	1.00	0.26
Catapodium rigidum	0.137	3.34	1.000	0.135	0.86	0.999	7-56	0.72	0.98	0.29
							7-120	0.96	0.97	0.27
Deschampsia flexuosa	0.101	0.55	0.996	0.079	0.62	0.994	7-56	0.77	0.84	0.19
							7-120	-0.81	1.20	0.65
Festuca ovina	0.113	1.62	0.995	0.095	0.76	0.993	7-56	0.80	0.86	0.18
							7-120	-0.47	1.02	0.33
Holcus lanatus	0.158	7.93	0.998	0.151	1.10	0.995	7-56	0.88	0.93	0.23
							7-120	0.96	0.87	0.19
Poa annua	0.165	21.1	0.997	0.154	1.83	0.993	7-56	0.80	0.91	0.20
							7-120	0.93	0.84	0.16
Cardamine hirsuta	0.153	2.87	0.976	0.115	0.27	0.982	7-56	0.71	0.78	0.06
							7-120	0.42	0.91	0.09
Centaurea nigra	0.134	3.05	0.993	0.134	2.06	0.997	7-56	-0.86	1.04	0.47
							7-120	-1.00	1.11	0.56
Rumex acetosa	0.136	7.02	0.993	0.143	2.48	0.991	7-56	-0.75	1.06	0.30
							7-120	-0.94	1.07	0.30
Scabiosa columbaria	0.130	14.7	0.998	0.119	11.0	0.999	7-56	0.69	0.94	0.19
							7-120	-0.93	1.15	0.30

Table S3 continued.

(b) Cooled plants

Species	Logistic models (eqn. (1)) fitted to data (Table S2)						Time interval (d)	Correlation between RMF and RGR	Allometry	
	Shoot			Root						
	r (d^{-1})	Y_{max} (g)	R^2	*r* (d^{-1})	Y_{max} (g)	R^2			α	β
Anthoxanthum odoratum	0.145	0.84	0.986	0.132	0.87	0.997	7-56	-0.68	1.03	0.48
							7-120	-0.95	1.25	0.88
Arrhenatherum elatius	0.157	0.84	0.973	0.155	0.50	0.982	7-56	-0.92	1.09	0.42
							7-120	-0.99	1.22	0.55
Catapodium rigidum	0.127	1.27	0.996	0.127	1.82	0.998	7-56	-0.36	1.02	0.36
							7-120	-0.99	1.27	0.90
Deschampsia flexuosa	0.102	0.21	0.993	0.081	0.68	0.998	7-56	-0.14	0.94	0.32
							7-120	-0.90	1.50	3.50
Festuca ovina	0.112	0.40	0.997	0.096	1.40	0.999	7-56	0.13	0.94	0.29
							7-120	-0.92	1.48	2.19
Holcus lanatus	0.153	1.46	0.967	0.161	0.96	0.981	7-56	-0.97	1.09	0.49
							7-120	-0.99	1.19	0.56
Poa annua	0.158	1.70	0.983	0.161	0.74	0.989	7-56	-0.98	1.04	0.38
							7-120	-0.97	1.10	0.38
Cardamine hirsuta	0.156	0.65	0.960	0.116	0.15	0.993	7-56	0.08	0.88	0.09
							7-120	-0.64	1.11	0.19
Centaurea nigra	0.130	1.02	0.991	0.134	1.18	0.998	7-56	-0.96	1.14	0.73
							7-120	-1.00	1.29	1.00
Rumex acetosa	0.135	1.15	0.981	0.150	0.34	0.980	7-56	-0.87	1.08	0.34
							7-120	-0.85	1.06	0.30
Scabiosa columbaria	0.127	0.63	0.981	0.115	0.47	0.995	7-56	-0.71	1.06	0.32
							7-120	-0.95	1.34	0.69
